# Comprehensive analysis of ECHDC3 as a potential biomarker and therapeutic target for acute myeloid leukemia: Bioinformatic analysis and experimental verification

**DOI:** 10.3389/fonc.2022.947492

**Published:** 2022-09-12

**Authors:** Yijing Zhao, Li-Ting Niu, Li-Juan Hu, Meng Lv

**Affiliations:** Peking University People’s Hospital, Peking University Institute of Hematology, National Clinical Research Center for Hematologic Disease, Beijing Key Laboratory of Hematopoietic Stem Cell Transplantation, Beijing, China

**Keywords:** ECHDC3, acute myeloid leukemia, immune cell infiltration, re-stratification, chemoresistance

## Abstract

**Background:**

Enoyl-CoA hydratase domain containing 3 (ECHDC3) increased in CD34^+^ progenitor cells of acute myeloid leukemia (AML) cells after chemotherapy. However, the prognostic significance and function of ECHDC3 in AML remain to be clarified.

**Methods:**

In the training cohort, 24 AML (non-acute promyelocytic leukemia, APL) patients were enrolled in Peking University People’s Hospital and tested for ECHDC3 in enriched CD34^+^ cells at diagnosis. In the validation set, 351 bone marrow RNA-seq data of non-APL AML were obtained by two independent online datasets (TCGA-LAML and BEAT-AML). LASSO regression model was conducted to a new prediction model of ECHDC3-related genes. In addition, the ECHDC3 signature was further explored by GO, KEGG, GSEA, and immuno-infiltration analysis. By RNA interference, the function of ECHDC3 in mitochondrial DNA (mt-DNA) transcriptome and chemoresistance was further explored, and the GSE52919 database re-verified the ECHDC3 chemoresistance feature.

**Results:**

By Kaplan-Meier analysis, patients with ECHDC3^high^ demonstrated inferior overall survival (OS) compared to those with ECHDC3^low^ both in the training (2-year OS, 55.6% *vs*. 100%, p = 0.011) and validation cohorts (5-year OS, 9.6% *vs*. 24.3%, p = 0.002). In addition, ECHDC3^high^ predicted inferior OS in the subgroup of patients with ELN 2017 intermediated (int) risk (5-year OS, 9.5% *vs*. 26.3%, p = 0.039) or FLT3+NPM1− adverse (adv) risk (4-year OS, 6.4% *vs*. 31.8%, p = 0.003). In multivariate analysis, ECHDC3 was an independent risk factor of inferior OS (HR 1.159, 95% CI 1.013–1.326, p = 0.032). In the prediction model combining ECHDC3 and nine selected genes (RPS6KL1, RELL2, FAM64A, SPATS2L, MEIS3P1, CDCP1, CD276, IL1R2, and OLFML2A) by Lasso regression, patients with high risk showed inferior 5-year OS (9.3% *vs*. 23.5%, p < 0.001). Bioinformatic analysis suggested that ECHDC3 alters the bone marrow microenvironment by inducing NK, resting mast cell, and monocyte differentiation. Knocking down ECHDC3 in AML cells by RNAi promoted the death of leukemia cells with cytarabine and doxorubicin.

**Conclusion:**

These bioinformatic analyses and experimental verification indicated that high ECHDC3 expression might be a poor prognostic biomarker for non-APL AML, which might be a potential target for reverting chemoresistance.

## Introduction

Acute myeloid leukemia (AML) is a heterogeneous disease characterized by different molecular subtypes with different prognoses and responses to treatment. Patients up to the age of 60 years reported 5-year survival rates of 30%–35% and <10%–15% for older patients (age 60 years and older) (1). The “3 + 7 regimen” (3 days of daunorubicin + 7 days of cytarabine) has long been considered the standard of care, resulting in long-term cures of 30%–40% among younger patients with AML. Therefore, finding a new target biomarker to change the therapy model from “one-set-fits-all” to “individualized stratified treatment” is an urgent problem needed to be solved (2).

Targeting mitochondrial metabolism was considered the most promising treatment for AML (3). Fatty acid metabolism is a key energy pathway for the survival of AML cells in the adipocyte-abundant bone marrow (BM) microenvironment. Adipocytes are the prevalent stromal cell type in adult BM, and leukemia cells continuously adapt to deficiency of nutrients acquiring chemoresistant profiles in the BM microenvironment (4). The latest data show that inhibition of fatty acid metabolism re-sensitizes resistant leukemia stem cells to venetoclax with azacitidine (5, 6). Enoyl-CoA hydratase domain containing 3 (ECHDC3), broadly expressed in adipocytes, is predicted to be active in the mitochondrion, involving fatty acid biosynthesis and lipid metabolism (7). Our team firstly reported that ECHDC3 was upregulated in CD34^+^ progenitors of chemoresistant AML (8), whereas the prognostic significance and function of ECHDC3 in AML have yet to be clarified.

By integrating bioinformatic analysis and experimental verification, this study aims to characterize the prognostic significance of ECHDC3 in AML and its role in chemoresistance and immune microenvironment.

## Materials and methods

### Patients and samples

According to the Declaration of Helsinki, the study was approved by the ethics review board of Peking University People’s Hospital, and enrolled patients provided written informed consent. All the enrolled patients were diagnosed and treated at Peking University People’s Hospital, Beijing, China.

The inclusion criteria are as follows: (i) newly diagnosed *de novo* AML (non-acute promyelocytic leukemia, APL), age 16–70 years; (ii) no previous antineoplastic therapy; (iii) ECOG performance score ≤ 2, without other major coexisting illnesses such as severe kidney and liver insufficiency.

BM samples were acquired before the first induction chemotherapy. Cell sorting and mass spectrometry measurements were followed as published study (8). The diagnosis, classifications, and risk stratification were based on European Leukemia Net (ELN) recommendations (9). Induction, consolidation, and allogeneic hematopoietic stem cell transplant (allo-HSCT) followed our previous report (10).

### Cell lines culture conditions

The human AML cell lines K562 were purchased from the American Type Culture Collection (ATCC) agent in China. All the cell lines have passed the STR authentication. Cell lines were cultured in RPMI 1640 medium (Thermo Fisher Scientific, Cat#11875093, USA) containing 10% FBS (Thermo Fisher Scientific, Cat#12483020, USA) and 1× antibiotic–antimycotic (Thermo Fisher Scientific, Cat#15240096, USA). Cells were grown at 37°C in a humidified atmosphere with 5% CO_2_.

### Online data acquisition

The clinical and transcription matrix of AML was downloaded from the TCGA database (https://portal.gdc.cancer.gov/) and the Beat AML database (http://www.vizome.org/aml/). We retrieved 154 non-APL AML BM mRNA sequence data (excluded 16 APL and 3 incomplete data) and corresponding clinicopathological characteristics from the TCGA. Additionally, 197 BM mRNA sequence data (excluded 9 APL and 25 incomplete data) were retrieved from the Beat AML database (BEAT-AML). The GSE52919 database (https://www.ncbi.nlm.nih.gov/geo/) was performed to analyze the correlation of ECHDC3 expression with cytarabine (Ara-C) sensitivity in AML samples.

### Lasso regression analysis, risk model establishment, and validation

Lasso regression was constructed by the glmnet R package according to the method previously published by our institute (11). In brief, 249 differentially expressed genes (DEGs) were identified by the comparison of ECHDC3 high and low expression samples according to the criteria ∣log_2_FC∣>1 and adjusted p < 0.05. LASSO regression was then applied to remove redundant prognostic genes based on the coefficient and partial likelihood deviance for developing the prognostic model. ECHDC3 and other Nine hub genes (RPS6KL1, RELL2, FAM64A, SPATS2L, MEIS3P1, CDCP1, CD276, IL1R2, and OLFML2A) were ultimately retained. Then, the hub genes were used to construct a risk signature based on the coefficient for each patient according to the following formula: $RS\;=\;\sum_{i=1}^{n}\;Coef\;\left(i\right)\;X\;\left(i\right)$ , where Coef(*i*) denotes the coefficient, and *X*(*i*) is the z-score transformed relative expression level for each DEG. To validate the established model, the prognostic risk score of each patient was calculated using the formula, and, according to the median score, patients were classified into high- and low-risk groups. Then, time-dependent receiver operating characteristic (ROC) curve (tdROC) and Kaplan-Meier survival curve analysis were performed to verify this risk score system (**Figures 4C, D**).

### Gene function analysis

Gene ontology (GO) and Kyoto Encyclopedia of Genes and Genomes (KEGG) enrichment analysis of differentially expressed ECHDC3 were performed using the R package “cluster profile”. GO terms were classified as molecular functions (MFs), biological processes (BP), and cellular components (CCs). Both p- and q-values less than 0.05 were considered to indicate statistically significant differences. Gene set enrichment analysis (GSEA) was performed to find enriched term differences.

### Immune infiltration analysis

The gene transcriptome data were used to estimate the content of multiple immune cells infiltrated in the BM microenvironment. CIBERSORT in combination with the LM22 signature matrix was used to estimate the fractions of 22 human immune cell phenotypes between AML samples with high or low ECHDC3 expression. The CIBERSORT analysis was conducted in R software by the CIBERSORT R script (version 1.03). The correlation analysis of the index was completed using the Spearman’s test.

### ECHDC3 knockdown

ECHDC3 siRNA was designed and synthesized by GenePharma (Shanghai, China). The plasmid was then transfected into the K562 cell lines. Opti-MEM medium and Lipofectamine L300015 reagent were used for the transfection of siRNA according to the manufacturer’s instructions. After transfection, whole-cell cDNAs were synthesized, and quantitative real-time PCR was performed to verify the ECHDC3 expression level.

### Cell viability assay

Ara-C (cytarabine) and doxorubicin (DOX) were purchased from Selleck Chemicals (Selleck, USA). Cell viability was determined by CCK-8 assay (Bimake, Cat#B34034, USA). The cells were seeded in a density of 2 × 10^4^ cells/100 μl in a 96-well plate and incubated at 37°C for 48 h. Ten microliters of CCK-8 was added to each well of the 96-well plate, followed by further incubation for 1 h. The absorbance was measured at 450 nm. The cell viability was calculated using GraphPad Prism software.

### Mitochondria transcriptome

Whole-cell cDNAs were synthesized, and quantitative real-time PCR was performed using the FastStart Universal SYBR Green Master mix (Millipore Sigma, MA, USA) with a StepOnePlus real-time PCR system (ABI Prism 7900HT; Applied Biosystems, USA). The threshold cycle (Ct) values of target genes were normalized over the Ct of β-actin in whole-cell lysate. For comparison, the siCT group was set as 1 unless otherwise indicated. Primers used for real-time PCR and quantitative real-time PCR are listed in **Table S1**.

### Statistical analysis

R software 4.05 and GraphPad Prism 8.0.2 were mainly used for laboratory data statistical analysis. Comparisons between the two groups were performed using the Mann-Whitney *U*-test for continuous variables and the X2 test for categorical data. P < 0.05 marked with *, P < 0.01 marked with **, and P < 0.005 marked with *** are considered significant. The survival functions were estimated by the Kaplan-Meier method using the log-rank test with asymmetric 95% confidence intervals (CIs). Variables included in the multivariate analysis (MVA) were selected by the backward elimination process with a criterion of P < 0.10 for retention. Age is linear with estimates of HRs for 10-year difference.

**Figure 1:**
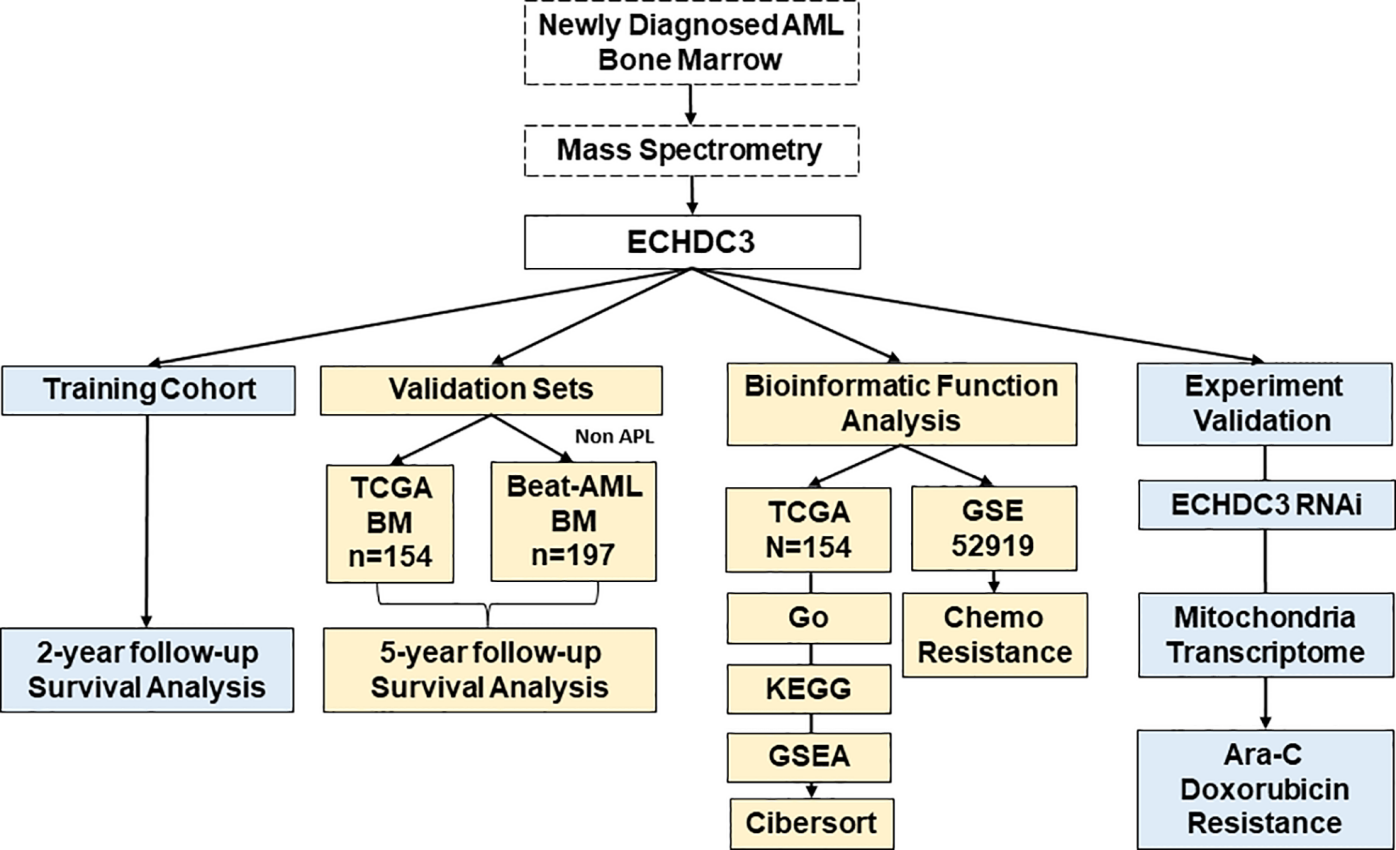
The flow chart of the study design and analysis.

The total study design is illustrated in **Figure 1**.

## Results

### High ECHDC3 predicts inferior OS in the training and validation cohort

**Figure 2:**
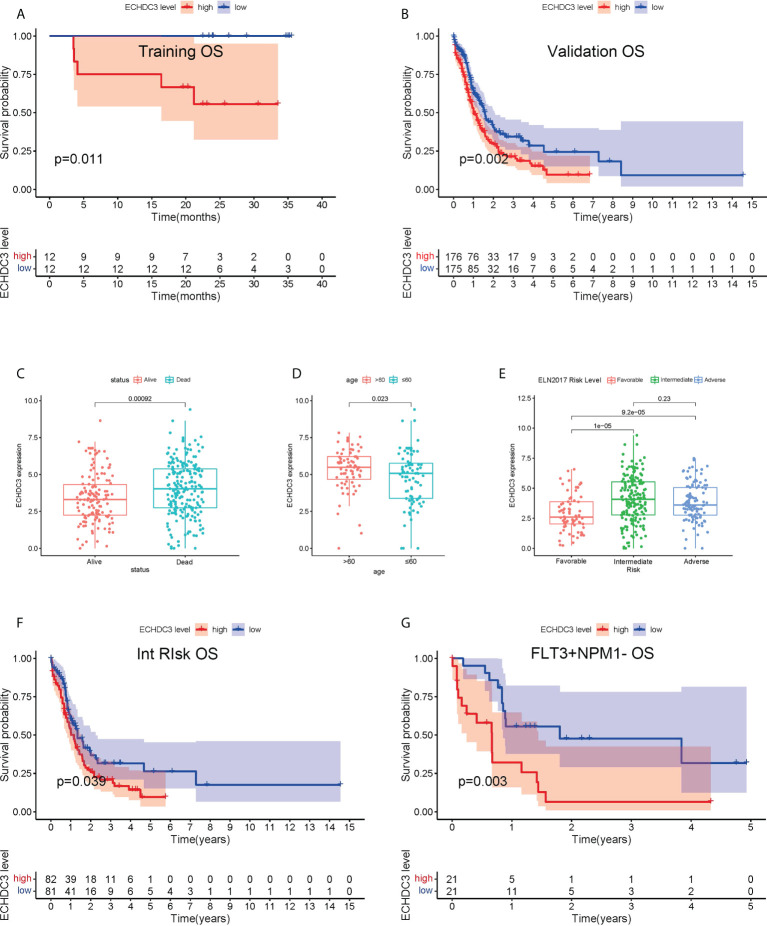
Prognostic value of ECHDC3 expression in training and validation. **(A)** Overall survival of non-APL AML patients in the training cohort (*de novo* AML patients). **(B)** Overall survival of non-APL AML patients in validation sets (TCGA-LAML and BEAT-AM bone marrow samples). **(C)** ECHDC3 expression in subgroups of alive and dead patients in validation sets. **(D)** ECHDC3 expression in subgroups of age >60 and age <=60 in validation sets. **(E)** ECHDC3 expression in subgroups of fav, int, and adv ELN risk in validation sets. **(F)** Overall survival of ELN int-risk AML in validation sets. **(G)** Overall survival of FLT3+NPM1− AML in validation sets.

In the training cohort, 24 AML (APL) patients were enrolled between September 2019 and September 2020, followed up to July 2022. According to the median protein level of ECHDC3 in enriched CD34^+^ cells at diagnosis, patients were divided into “ECHDC3^high^” and “ECHDC3^low^” groups, and the baseline characteristics were similar between the two groups (**Table S2**). By median follow-up of 23.7 months, 11 patients experienced relapse, which corresponds to a 2-year probability of relapse at 45.8% (95% CI, 24.1–65.1), in which 5 patients achieved CR2 and preceding to allo-HSCT. None of these patients had non-relapse mortality. OS was significantly lower in the ECHDC3^high^ group than in the ECHDC3^low^ (2-year OS, 55.6% *vs*. 100%; 95% CI, 23.1–79.0 *vs*. 100%; p = 0.011, **Figure 2A**). There was a trend that ECHDC3^high^ conferred inferior leukemia-free survival (2-year LFS, 41.7% *vs*. 66.7%, p = 0.178, **Figure S1A**) but without statistical significance.

In the validation set, 351 BM RNA-seq data of non-APL AML were obtained by two independent online datasets (TCGA-LAML and BEAT-AML). Patients were divided into “ECHDC3^high^” and ECHDC3^low^” groups according to the median expression level of ECHDC3 in BM, and the baseline characteristics were listed in **Table 1**. OS was significantly lower in the ECHDC3^high^ group than in the ECHDC3^low^ (5-year OS, 9.6% *vs*. 24.3%; p = 0.002; **Figure 2B**). Correspondingly, the alive patients showed a lower ECHDC3 expression level compared to dead patients (P = 0.0009; **Figure 2C**). Of the RNA-seq data of 180 peripheral blood (PB) samples of non-APL AML in BEAT-AML, ECHDC3^high^ also showed inferior 5-year OS (3.5% *vs*. 14.8%; 95% CI, 23.1–79.0 *vs*. 100%; p = 0.045; **Figure S1B**).

**Table 1:** Patient characteristics of validation sets.

	ECHDC3 High (n =176)	ECHDC3 Low (n = 175)	P
Sex, male/female	100/76	94/81	0.4898
Median age, years (range)	61 (21–88)	42.5 (18–61)	<0.0001*
Median PB blasts, %	67 (0–99)	47 (0–99.2)	0.0004*
Median WBC, × 109/L	22.95 (1–297)	17.9 (0.5–427.46)	0.0724
Median BM blasts, % (range)	36 (0–98)	62 (0–98)	<0.0001*
FAB classifications			<0.0001*
M0	13	6	
M1	30	18	
M2	30	12	
M4	34	17	
M5	26	14	
M6	2	0	
M7	3	1	
NA	38	107	
Risk level			0.1714
Favorable	22	48	
Intermediate	102	71	
Adverse	49	55	
NA	3	1	

*means statistic difference was observed.

NA, Not Available.

In the validation set, the expression of ECHDC3 was analyzed by age and ELN risk groups. Patients more than 60 showed higher ECHDC3 levels compared to those less or equal to 60 (p = 0.023; **Figure 2D**). In different risk subgroups, patients of int risk or adv risk showed a higher level of ECHDC3 compared with those of favorable (fav) risk (p < 0.001; p < 0.001; respectively; **Figure 2E**); meanwhile, there was no significant difference in ECHDC3 level between int- and adv-risk groups (p=0.23).

Furthermore, to test whether ECHDC3 would be a useful biomarker in re-stratification of AML patients, subgroup analysis was carried out in fav-, int-, and adv-risk AML, as well as in patients with or without NPM1/FLT3 mutation. ECHDC3^high^ predicted inferior 5-year OS in the subgroup of patients with ELN int-risk (9.5% *vs*. 26.3%, p = 0.039; **Figure 2F**) but not in the fav-risk or adv-risk groups (**Figures S1C, D**). In addition, ECHDC3^high^ predicted inferior 5-year OS in the subgroup of FLT3+NPM1− adv-risk AML (6.4% *vs*. 31.8%, p = 0.003; **Figure 2G**). There was a trend that ECHDC3^high^ related to inferior 3-year OS of FLT3−NPM1+ fav-risk AML (31.7% *vs*. 54.8%, p = 0.077, **Figure S1E**) but without significance. Meanwhile, ECHDC3 did not affect OS in FLT3−NPM1− or FLT3+NPM1+ AML patients (**Figures S1F, G**).

As an expression of ECHDC3 in BM was associated with a high percentage of blast cells in PB and a low percentage of blast cells in BM (**Figures S1H**; **Table 1**), while there was a trend that a high percentage of blast cells in PB rather than BM might be associated with inferior OS (**Figure S1I, J**), subgroup analysis was carried out addressing the percentage of blast cells in PB, which indicated that ECHDC3^high^ would be a useful biomarker predicting 5-year OS in the subgroup with a high (p < 0.001) rather than a low percentage of blast cells (p = 0.537) in PB (**Figures S1K, L**).

**Figure 3:**
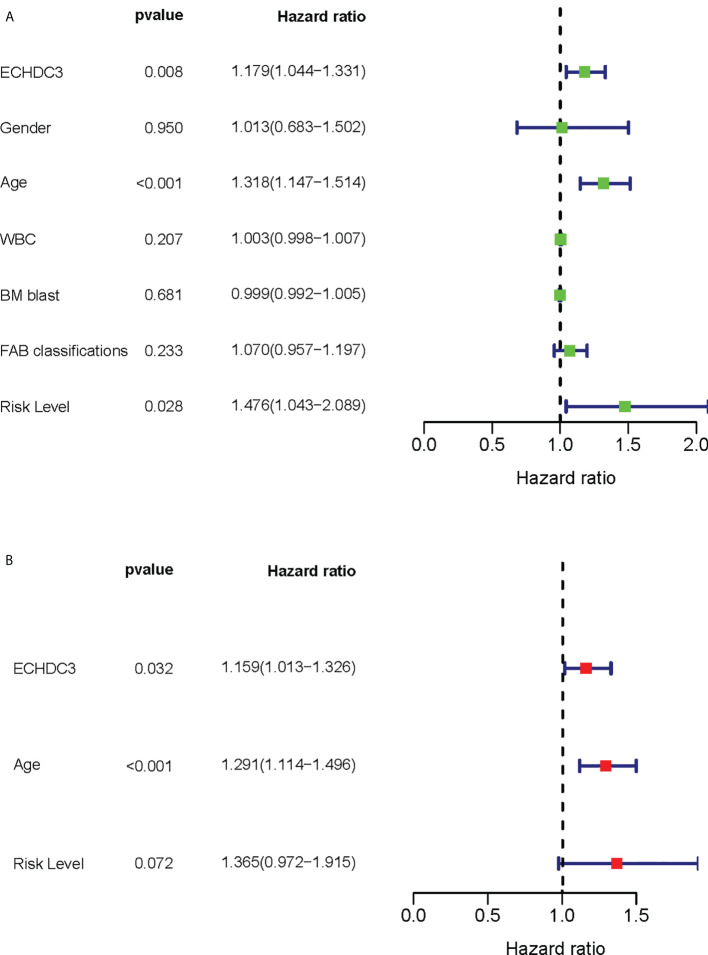
Cox regression analysis of ECHDC3 expression in validation sets. **(A)** Univariate Cox regression analysis for identification of AML patients’ clinical character in validation sets. **(B)** Multivariate Cox regression analysis for identification of AML patients’ clinical character in validation sets.

Among the confounding factors in univariate analysis, ECHDC3, age, and risk stratification level were chosen for MVA (**Figure 3A**). ECHDC3 was confirmed as an independent prognostic factor for OS (HR 1.159, 95% CI 1.013–1.326, p = 0.032; **Figure 3B**). Age is another independent prognostic factor (HR 1.291, 95% CI 1.114− 1.496, p < 0.001). Meanwhile, ELN risk might be an independent prognostic factor without statistical significance (HR 1.365, 95% CI 0.972−1.915, p = 0.072; **Figure 3B**).

### The combination of ECHDC3 and other risk factors improves the accuracy of survival prediction

**Figure 4:**
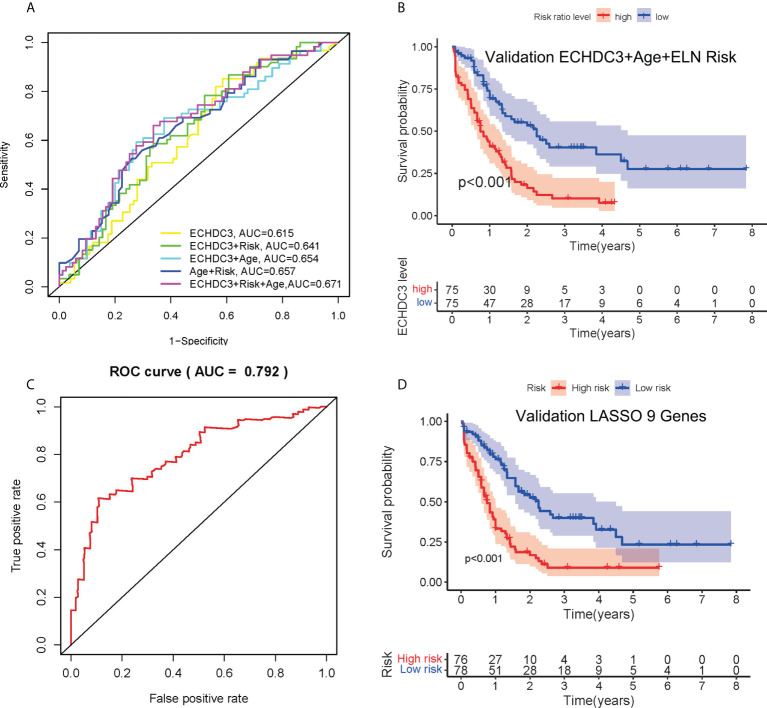
Combination of ECHDC3 and other risk factors in the prediction. **(A)** Time-dependent ROC analysis by combination of ECHDC3+age+ELN risk **(B)** Overall survival comparing high *vs*. low risk in the new model of ECHDC3 + age + ELN risk. **(C)** Time-dependent ROC analysis in the new model by LASSO regression. **(D)** Overall survival comparing high *vs*. low risk in the new model by LASSO regression.

As ECHDC3, age, and ELN risk were independent risk factors for OS, then we combined these three factors as a new prognostic model and tested it in non-APL AML patients of TCGA-LAML datasets (excluding four cases due to missing data). Adding ECHDC3 expression to age and ELN risk improved the area under the curve (AUC) from 0.657 to 0.671 of ROC (**Figure 4A**). A risk-score model combining ECHDC3, age, and ELN risk was developed based on a linear combination of each parameter (Coef) weighted by the regression coefficient derived from the univariate Cox regression analysis (β). The equation was calculated as $RS\;=\;\sum_{i\;=\;1}^{154}\;Coef\;\left(i\right)\;*\text{ β }\left(i\right)$ .According to the median risk score, patients with high-risk scores have inferior 5-year OS (7.6% *vs*. 27.7%, p < 0.001; **Figure 4B**).

In non-APL AML patients of TCGA-LAML datasets, a total of 249 survival correlated genes (according to the criteria ∣log2FC∣ > 1 and adjusted p < 0.05) performed 6-fold cross-validation and 9 genes in addition to ECHDC3 were finally found with regression coefficients, namely, RPS6KL1, RELL2, FAM64A, SPATS2L, MEIS3P1, CDCP1, CD276, IL1R2, and OLFML2A (**Figures S2A–C**). Each gene was an independent prognostic factor by two-way interactions checked between the factors with the main effect in chi-square analysis (**Figure S2D**). Next, the prognostic model was established with Lasso regression to improve the predicted accuracy for overall survival in AML. The equation is calculated as $RS\;=\;\sum_{i=1}^{154}\;Coef\;\left(i\right)\;*\;gene\;expression\;\left(i\right)$ , and that is Y = ECHDC3 × 0.0127-RPS6KL1 × 0.0006 + RELL2 × 0.0256-FAM64A × 0.0823 + SPATS2L × 0.0611-MEIS3P1 × 0.0284 + CDCP1 × 0.0296 + CD276 × 0.0099 + IL1R2 ×0.0033 + OLFML2A × 0.1069 in this study. Y ≥ 3.090 is categorized as “high risk” (**Figures S2E–F**). The AUC of the ROC curve following this risk model is 0.792 (**Figure 4C**). Patients with high-risk score have inferior 5-year OS (9.3% *vs*. 23.5%, p < 0.001; **Figure 4D**).

### Bioinformatic analysis of ECHDC3 function

**Figure 5:**
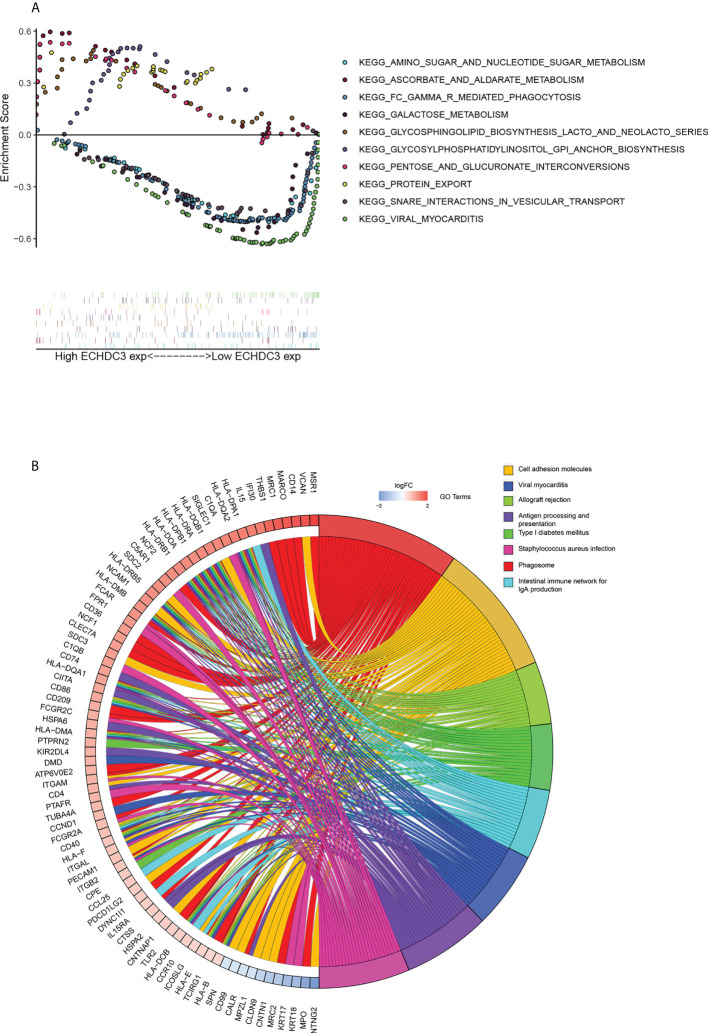
Bioinformatic analysis of ECHDC3 function. **(A)** Enriched KEGG pathways in ECHDC3^high^ and ECHDC3^low^ AML patients by GSEA. **(B)** GO Chord plot: ECHDC3 differentially expressed genes were linked *via* ribbons to the assigned terms, blue-to-red coding indicated logFC.

GSEA was performed to identify numbers of hallmark terms enriched in the high ECHDC3_exp and low ECHDC3_exp groups. The top 6 Hallmark terms identified in the low ECHDC3_exp group were mainly immune-related (**Figure S3A**), namely, complement system, IL6 signaling, IFN-α, IFN-γ, P53, and xenobiotic metabolism. Additionally, **Figure 5A** showed the KEGG term enriched in high ECHDC3_exp and low ECHDC3_exp groups.

The top 40 DEGs, including 20 upregulated genes and 20 downregulated genes in ECHDC3^high^ and ECHDC3^low^ groups, were demonstrated in the heatmap (**Figure S3B**). By comparison of the top KEGG pathways enriched in DEGs in ECHDC3^high^ and ECHDC3^low^ groups, it showed that these marker genes mainly enriched in the phagosome, the intestinal immune network for IgA production, cell adhesion molecules, viral myocarditis, viral myocarditis, allograft rejection, antigen presentation, type I diabetes mellitus, and staphylococcus aureus infection (**Figure S3C**). GO analysis further declared the potential function of the DEGs (**Figure S3D**). The DEGs were most significantly enriched in the BP term of “positively regulation of cytokine production” and “regulation of leukocytes proliferation”, in addition to the CC term of “collagen-containing extracellular matrix” and “external side of plasma membrane’’. In MF term, genes were enriched of “amide binding” and “peptide binding”. These results indicated that the DEGs were distributed in the immune system–related processes and pathways, which have been proved to play a pivotal role in the immune microenvironment. The top 8 enriched GO terms and demonstrated represents genes were demonstrated in **Figure 5B**.

### The immune landscape between ECHDC3^high^ and ECHDC3^low^ groups

**Figure 6:**
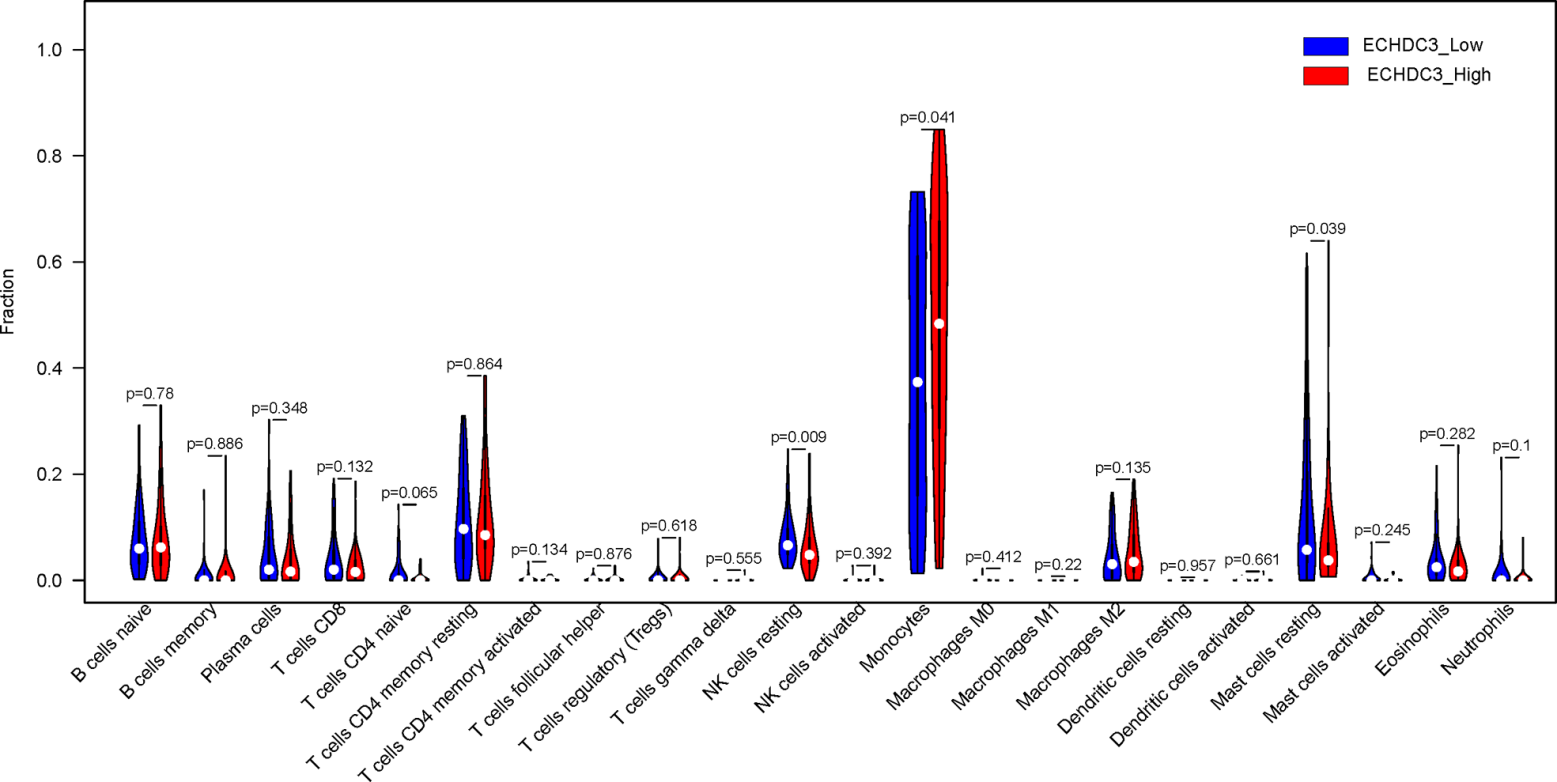
Different immune cells between ECHDC3^high^ and ECHDC3^low^ AML patients.

In the BM microenvironment, immune infiltrating cells controlled the fate of AML cells. In this study, the composition of significant tumor-infiltrating immune cells was assessed by the CIBERSORT algorithm in the AML BM RNA-seq dataset. The histogram of immune infiltrating cells was shown in **Figure S4A**. Moreover, resting NK cells (p = 0.009), monocytes (p = 0.041), naïve CD4^+^ T cells (p = 0.065), and resting mast cell (p= 0.039) had significant differences in the immune cell fractions between the two groups ECHDC3^high^ and ECHDC3^low^ (**Figure 6**). Next, the co-expression analysis using prognostic tumor-infiltrating immune cells in the AML cohort with ECHDC3^high^ and ECHDC3^low^ was performed. From **Figures S4B–C**, we can see that monocytes have a negative correlation with resting CD4^+^ T memory cells and CD8^+^ T cells in the immune phenotype profiles, whereas resting NK cells were positively correlated with T regulatory cells and resting CD4^+^ T memory cells. Considering all the interpreted results, higher ECHDC3 expression may induce NK, mast cell resting, and monocyte differentiation, and ECHDC3 may directly alter the BM microenvironment cell infiltration.

**Figure 7:**
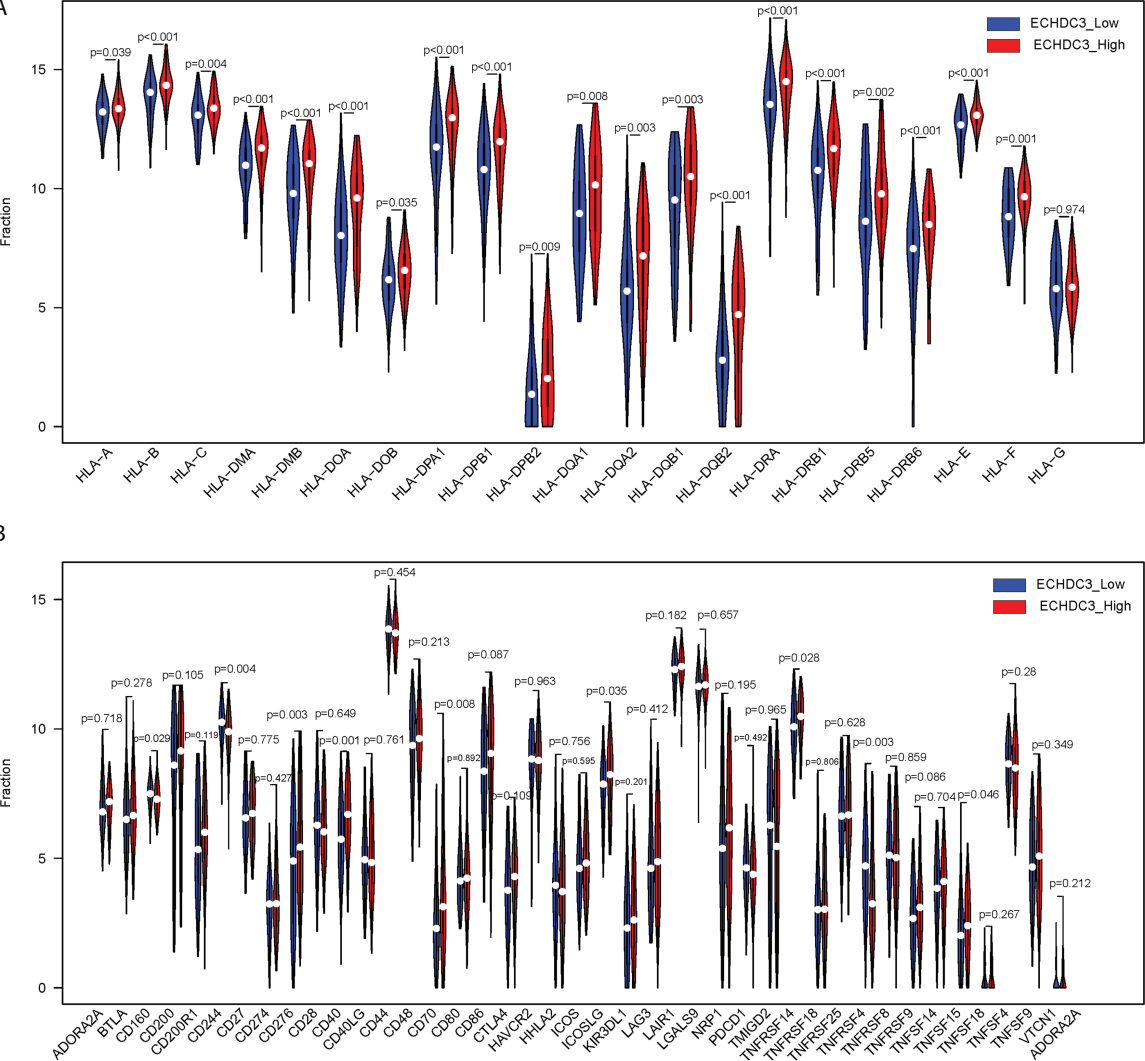
Comparison of HLA gene expression and immune checkpoint gene expression between ECHDC3 high and low subgroup. **(A)** HLA gene expression in ECHDC3 high and low subgroups. **(B)** Immune checkpoint gene expression in ECHDC3 high and low subgroups.

The human leukocyte antigen (HLA) system is an important part of the immune system, and HLA encodes cell surface molecules specialized to present antigenic peptides to the T-cell receptor (TCR) on T cells (12). Immune checkpoints are regulators of the immune system. These pathways are crucial for self-tolerance, which prevents the immune system from attacking cells indiscriminately. However, some cancers can protect themselves from attack by stimulating immune checkpoint targets (13). The expression information of the HLA family and immune checkpoints was shown (**Figures 7A, B**), which indicated that the HLA family and immune checkpoints were significantly different between the ECHDC3^high^ and ECHDC3^low^ groups. As can be seen in **Figure 7B**, TNF superfamily member 15 (TNFSF15) and CD40 were significantly upregulated in the ECHDC3^high^ subgroup with p < 0.001, whereas TNF superfamily member 4 (TNFSF4) were significantly downregulated (p = 0.003).

### ECHDC3 promote chemoresistance

**Figure 8:**
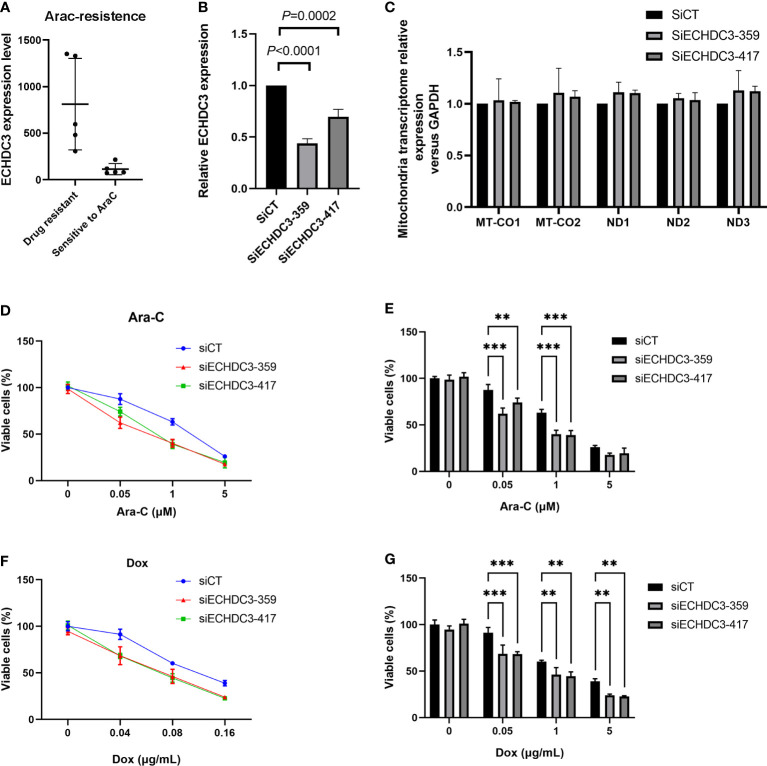
ECHDC3 in chemoresistance. **(A)** Higher ECHDC3 promotes chemoresistance by analyzing the GSE52919 database. **(B)** Knocking down ECHDC3 by RNAi. **(C)** Comparison of mt-DNA transcriptome by knocking down ECHDC3. **(D, E)** Cell viability treated with Ara-C. **(F, G)** Cell viability treated with doxorubicin. ***P<0.001, **P<0.01.

In the GSE52919 database, patients with Ara-C resistance showed higher ECHDC3 expression compared with sensitive patients (p = 0.0136; **Figure 8A**).

To verify the role of ECHDC3 in mt-DNA transcriptome and chemoresistance by experiments, ECHDC3 was knocked down by RNAi (SiECHDC3-351 and SiECHDC3-417) in the K562 cell lines (**Figure 8B**). The mt-DNA transcriptomes (MT-CO1, MT-CO2, ND1, ND2, and ND3) did not alter by inhibiting ECHDC3 (**Figure 8C**), whereas knocking down of ECHDC3 promoted the death of leukemia cells in either Ara-C at 0.1–1 µM or DOX at 0.04–0.16 µg/ml (**Figures 8D–G**).

## Discussion

Previous studies about ECHDC3 were mainly focused on its role in Alzheimer’s disease (14), acute coronary syndrome (15), and acute peanut allergic reactions (16), rather than on hematological malignancies. By extension of our previous finding of changed ECHDC3 expression in chemoresistant AML, the current study made a comprehensive analysis with bioinformatic analysis and experimental verification, supporting ECHDC3 as a novel biomarker predicting poor-prognosis AML, which might further improve risk stratification in AML subgroups. Both bioinformatic analysis and experiment supported the role of ECHDC3 in mediating chemoresistance, and bioinformatic analysis suggested that ECHDC3 alters the BM microenvironment, which warranted further exploration of ECHDC3 in AML in near future.

The present paper presents a set of evidence for proving that ECHDC3 is a poor prognostic biomarker and a therapeutic target of AML through proteomics, genomics, and mitochondrial transcriptomics. Improvement in AML patient stratification is critical in clinical practice. Next-generation sequencing technologies are gradually implemented in the clinical setting to facilitate diagnosis and treatment decisions in AML. Currently, ELN 2017 criteria have been widely applied as the standard risk stratification method to guide treatment in the first complete remission period in AML (17), and the latest ELN 2022 recommendation has just been published (18). In the present study, we firstly demonstrated the expression of ECHDC3 as an independent prognostic factor in addition to the current risk stratification system. As the expression of ECHDC3 is related to patients’ age (>60 *vs*. ≤60) as well as to ELN risk stratification (int/adv *vs*. fav), the question of whether ECHDC3 would be an independent biomarker is raised. Firstly, it is demonstrated that ECHDC3 and age remained the only two independent risk factors for inferior OS in MVA. Secondly, a new prediction model established by integrating ECHDC3 with basic characteristics including age and ELN risk groups further improved the accuracy of survival prediction, which highlighted the possibility of integration of ECHDC3 as an independent risk factor into risk stratification system in the future. Nevertheless, the current study did not follow the latest ELN 2022 criteria; therefore, the meaning of ECHDC3 in AML under ELN 2022 criteria is needed to be further validated by a larger cohort in the future.

Moreover, because allo-HSCT is generally considered a favorable consolidation treatment option in adv-risk AML, whereas chemotherapy is the preferred therapy in fav AML in the absence of persisting measurable residual diseases (MRD), it remained controversial whether allo-HSCT should be recommended as a priority in patients with int-risk AML. Several groups addressed this question by adding other gene profiles to the ELN 2017 criteria. Hu et al. established a prognostic nomogram characterized by white blood cell count (≥10×10^9^/L at diagnosis), mutated DNMT3A, and genes involved in signaling pathways, which divided int AML into two subgroups that could predict that the high-risk subgroup was associated with inferior OS and relapse-free survival (RFS), whereas allo-HSCT reduced the relapse risk of high-risk patients (3-year RFS: allo-HSCT: 40.0 *vs*. chemotherapy, P = 0.010) (19). Eisfeld et al. refined ELN 2017 by adding mutated BCOR, mutated SETBP1, and mutated IDH2 and removed NPM1mut with FLT3^high^ in int AML; the outcome of 131 patients in the new int-risk group improved in comparison with that of the original int-risk group (n = 189) concerning both DFS (3-year rates: 32% *vs*. 22%) and OS (3-year rates: 41% *vs*. 31%) (20). ELN 2022 re-classified FLT3+ with mutation or wild-type NPM1 as int risk instead of previous adv risk (18). Therefore, it is important to further divide int-risk AML into subgroups with diverse relapse risks. In the present study, the expression of ECHDC3 could divide int-risk AML and FLT3+NPM1+AML into two subgroups with distinct survival rates, which might improve the prediction of the survival and guide post-remission treatment in int-risk AML.

Considering the negative effect of high ECHDC3 expression on the survival of AML patients, it becomes an important question why and how ECHDC3 promotes AML. By bioinformatic analysis and experimental verification, the current study preliminarily supported that ECHDC3 might be associated with chemoresistance in AML treatment. Although ECHDC3 was predicted to be active in the mitochondrion, knocking down ECHDC3 in the current study did not change the mt-DNA transcriptome, which remained to be explored in the future.

It is critical to develop a novel immune therapy targeting the BM microenvironment of AML. As important cytotoxic and cytokine-producing components of the innate immune system, NK cells might become key effector cells in immunotherapy for AML. Our group demonstrated that expanded clinical-grade membrane-bound IL-21/4-1BBL NK cell products could exhibit activity against AML *in vivo* (21). However, in the AML immune microenvironment, there are prominent immunosuppressive barriers, which result in dysfunction and exhaustion of NK cells (22). Resting NK cells are generally less lytic against target cells than *in vitro* interleukin 2 (IL-2)–activated NK cells. It has been revealed that the NK function is related to the levels of the fatty acids, and treatment of NK cells with saturated fatty acids or arachidonic acid significantly enhanced killing receptor expressions and decreased inhibitory NK (KIR) receptors, whereas docosahexaenoic and eicosapentaenoic acids increased KIR (23). For the first time, we found that the BM microenvironment immune phenotype was related to the fatty acid metabolism gene ECHDC3, which might contribute to its negative effect on the survival of AML patients by inhibiting NK function, which remained to be explored *in vivo* and *in vitro* research in near future.

In addition, monocyte differentiation is also of great importance in the immune microenvironment of AML, especially considering its closing relationship with monocytic myeloid-derived suppressor cells (M-MDSCs). Immature myeloid cells are characterized by the ability to suppress immune responses and expand during cancer (24). As M-MDSCs are morphologically and phenotypically similar to monocytes, it is the immune suppression that allows MDSCs to be distinguished from other myeloid cell populations. Coculture of the AML cell lines or primary AML cells with donor PB mononuclear cells expanded M-MDSCs and prevented monocyte differentiation, probably by MUC1-mediated tumor-derived extracellular vehicles (25). In clinical studies, it has been reported that tumor-activated ILC2s secreted IL-13 to induce M-MDSCs and support tumor growth, whereas ATRA treatment reversed the increase of ILC2-MDSCs in AML (26). The intensity of the immune response triggered by ECHDC3 may help us understand the immune microenvironment of AML.

## Conclusion

In sum, this study firstly described ECHDC3 as a negative AML prognosis predictor, which might refine the risk stratification of AML, especially in int AML. ECHDC3 is also involved in chemoresistance and shaping the immune microenvironment of AML, which could be a potential target for chemoresistance reverting and immune microenvironment remodeling for AML patients in the future.

## Data availability statement

The original contributions presented in the study are included in the article/**Supplementary Material**. Further inquiries can be directed to the corresponding authors.

## Ethics statement

This study was reviewed and approved by the ethics review board of Peking University People’s Hospital (2022PHB030). The patients provided their written informed consent to participate in this study.

## Author contribution

YZ, L-JH, and ML participated in the study design. YZ and L-TN participated in experiments. YZ and ML analyzed the data and wrote the manuscript. All authors contributed to the article and approved the submitted version.

## Funding

This work was supported by: The national key research and development plan of China (2021YFA1100902), National Natural Science Foundation of China (Grant No. 82100168, 82070182, 82070181), Beijing Nova Program of Science and Technology (No. Z211100002121058, Z191100001119120), Peking University People’s Hospital Research and Development Funds (RS2020-03, RDY2020-29), Peking University Medicine Fund of Fostering Young Scholars’ Scientific and Technological Innovation and supported by “the Fundamental Research Funds for the Central Universities” (BMU2021PYB005), China Scholarship Council (202106015007).

## Acknowledgments

We appreciated Dr. Xingchen Li (Department of Obstetrics and Gynecology, Peking University People’s Hospital) providing help in the data analysis.

## Conflict of interest

The authors declare that the research was conducted in the absence of any commercial or financial relationships that could be construed as a potential conflict of interest.

## Publisher’s note

All claims expressed in this article are solely those of the authors and do not necessarily represent those of their affiliated organizations, or those of the publisher, the editors and the reviewers. Any product that may be evaluated in this article, or claim that may be made by its manufacturer, is not guaranteed or endorsed by the publisher.

## Supplementary material

Supplementary Figure 1 Prognostic value of ECHDC3 expression in specific subgroups **(A)** Leukemia-free survival of non-APL AML patients in training cohort (*de novo* AML patients) **(B)** Overall survival of non-APL AML patients in validation sets (TCGA-LAML and BEAT-AM peripheral blood samples) **(C)** Overall survival of fav-risk AML patients in validation sets **(D)** Overall survival of adv-risk AML patients in validation sets **(E)** Overall survival of FLT3−NPM1+ AML patients in validation sets **(F)** Overall survival of FLT3−NPM1− AML patients in validation sets **(G)** Overall survival of FLT3+NPM1+ AML patients in validation sets **(H)** Distribution of Blast cells in PB or BM in validation sets **(I)** Overall survival of non-APL AML patients in validation sets according to PB blast **(J)** Overall survival of non-APL AML patients in validation sets according to BM blast **(K)** Overall survival of patients in validation sets with high PB blast **(L)** Overall survival of patients in validation sets with low PB blast.

Supplementary Figure 2 LASSO regression **(A)** The interrelation of 4 key genes acquired from LASSO regression **(B)** The evaluation progress of gene selection in LASSO regression **(C)** Heatmap of 9 key genes expression acquired from LASSO regression **(D)** Two-way interactions checked between the factors with the main effect in Lasso regression.

Supplementary Figure 3 Bioinformatic analysis of ECHDC3 function **(A)** Hallmark gene sets enrichment analyses for ECHDC3^high^ and ECHDC3^low^ AML patients by GSEA. **(B)** Heatmap of the deferentially expressed gene related to ECHDC3 expression. **(C)** KEGG Cluster plot displaying a circular dendrogram of the clustering of the ECHDC3 expression profiles. The inner ring shows the colored logFC, and the outer ring is assigned functional terms. **(D)** Identification of AML subtype-specific GO terms. Top 10 GO terms enriched in the Biological Process (BP), Molecular Function (MF), and Cellular Component (CC).

Supplementary Figure 4 Immune landscape between ECHDC3^high^ and ECHDC3^low^ groups **(A)** The heat map visualized the percentage abundance of tumor-infiltrating immune cells in each sample. **(B)** Immune cell infiltration correlation matrix. **(C)** Correlation matrix of all 22 immune cell proportions.
