# Supplementary material for: Comprehensive analysis of ECHDC3 as a potential biomarker and therapeutic target for acute myeloid leukemia: Bioinformatic analysis and experimental verification
Source: Front Oncol. 2022 Sep 12;12:947492. doi: 10.3389/fonc.2022.947492 (PMC9511173; doi:10.3389/fonc.2022.947492)
Supplement: Supplementary file 1 [file Table_1.docx]

Table S1 Primer sequence of RNAi and Realtime PCR

| ID | Primer Name | Primer sequence**(5'to3')** |
| --- | --- | --- |
| JH5809 | mtND1-F1 (5’-primer for human mitochondria ND1, 125bp) | GCATACCCCCGATTCCGCTA |
| JH5810 | mtND1-R1 (3’-primer for human mitochondria ND1, 125bp) | TGAGGGGGAATGCTGGAGAT |
| JH3293 | mtND2-F1 (5’ primer for human mitochondria ND2, 153bp) | CATGCTAGCTTTTATTCCAGTTC |
| JH3294 | mtND2-R1 (3’ primer for human mitochondria ND2, 153bp) | GTTATGGTTCATTGTCCGGAGAGT |
| JH5811 | mtND3-F1 (5’-primer for human mitochondria ND3, 125bp) | CGAGTGCGGCTTCGACCCTA |
| JH5812 | mtND3-R1 (3’-primer for human mitochondria ND3, 125bp) | AGGGGTAAAAGGAGGGCAATTTC |
| JH3289 | mtCox1-F1 (5’ primer for human mitochondria Cox1, 172bp) | CGCTATCCCCACCGGCGTCAAAG |
| JH3290 | mtCox1-R1 (3’ primer for human mitochondria Cox1, 172bp) | GTCGTGTAGTACGATGTCTAGTG |
| JH3291 | mtCox2-F1 (5’ primer for human mitochondria Cox2, 134bp) | ATGGCACATGCAGCGCAAGTAGGTC |
| JH3292 | mtCox2-R1 (3’ primer for human mitochondria Cox2, 134bp) | GTTAGGAAAAGGGCATACAGGAC |
| YJ001 | ECHDC3-1-forward(183bp) | AACGTCGGGCTCTTCTGTTC |
| YJ002 | ECHDC3-1-reverse(183bp) | CTGGGCAGAACGTCCTGATA |
| YJ003 | ECHDC3-2-forward(185bp) | CATATCAGGACGTTCTGCCCA |
| YJ004 | ECHDC3-2-reverse(185bp) | ATCGGCAAACCTACCCAGTG |
| J880 | Actin-R(135 bp) | CAGGTCATCACCATTGGCAATGAGC |
| J881 | Actin-F(135 bp) | CGGATGTCCACGTCACACTTCATGA |
| SiCT-1 | Negative control-R | UUCUCCGAACGUGUCACGUTT |
| SiCT-2 | Negative control-R | ACGUGACACGUUCGGAGAATT |
| YJ359S | ECHDC3-HOMO-359-S | GCAUAAGGAACAUCGUCUUTT |
| YJ360AS | ECHDC3-HOMO-359-AS | AAGACGAUGUUCCUUAUGCTT |
| YJ417S | ECHDC3-HOMO-417-S | GCUGAAGUCUCUCCAAAGUTT |
| YJ417AS | ECHDC3-HOMO-417-AS | ACUUUGGAGAGACUUCAGCTT |

Table S2 Patient characteristics of Training Cohort

|  | ECHDC3  High (n =12) | ECHDC3  Low (n = 12) | P |
| --- | --- | --- | --- |
| Sex, male/female | 5/7 | 7/5 | 0.6843 |
| Median age, years (range) | 37 (30-68) | 36(25-68) | 0.7011 |
| FAB classifications |  |  | 0.4783 |
| M0 | 0 | 1 |  |
| M2 | 12 | 9 |  |
| M4 | 0 | 2 |  |
| Risk level |  |  | 0.9667 |
| Favorable | 3 | 3 |  |
| Intermediate | 8 | 7 |  |
| Adverse | 1 | 2 |  |
