# Supplementary figures and images for: Comprehensive analysis of ECHDC3 as a potential biomarker and therapeutic target for acute myeloid leukemia: Bioinformatic analysis and experimental verification
Source: Front Oncol. 2022 Sep 12;12:947492. doi: 10.3389/fonc.2022.947492 (PMC9511173; doi:10.3389/fonc.2022.947492)

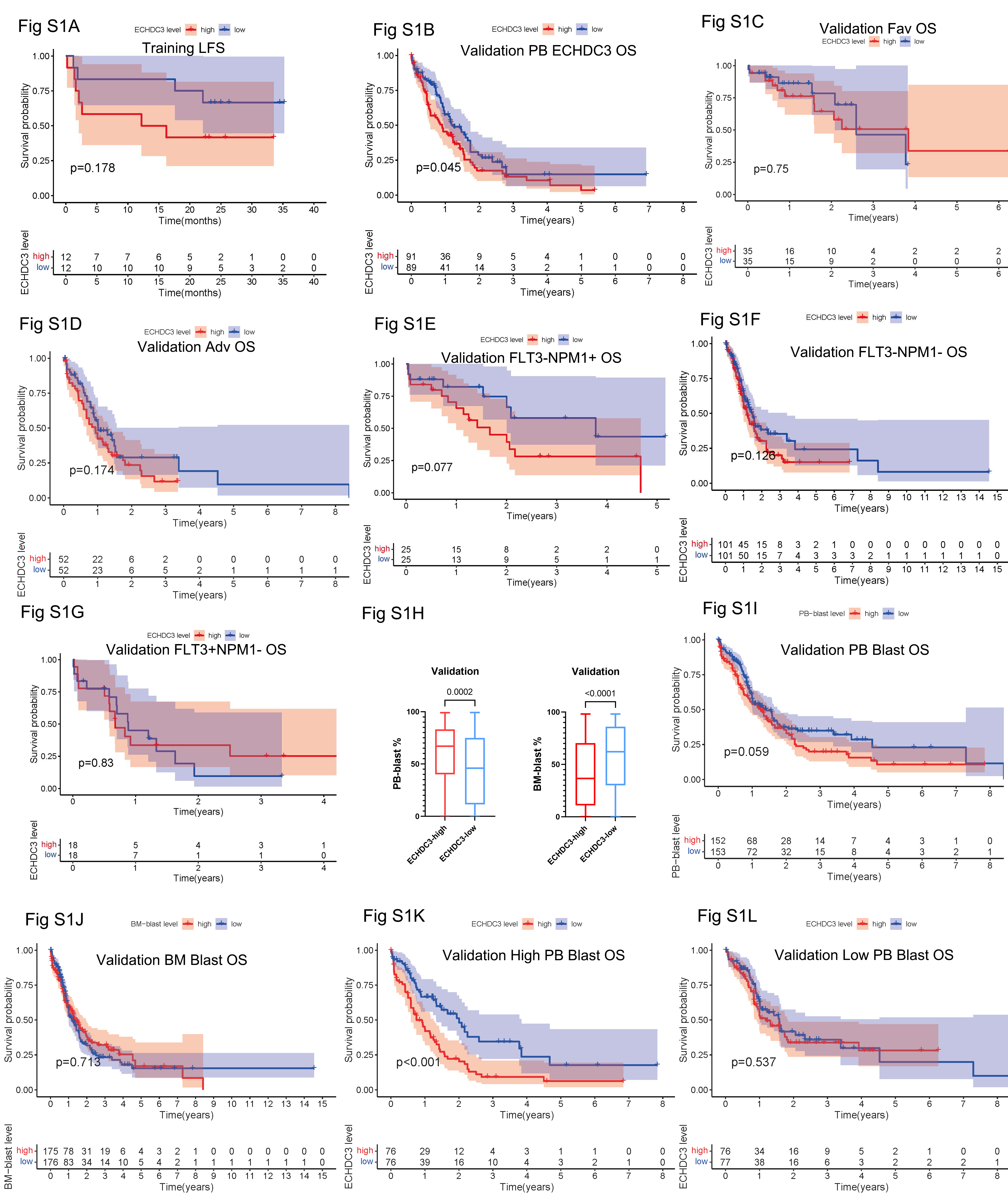

Supplement: Supplementary Figure 1 — Prognostic value of ECHDC3 expression in specific subgroups (A) Leukemia-free survival of non-APL AML patients in training cohort (de novo AML patients) (B) Overall survival of non-APL AML patients in validation sets (TCGA-LAML and BEAT-AM peripheral blood samples) (C) Overall survival of fav-risk AML patients in validation sets (D) Overall survival of adv-risk AML patients in validation sets (E) Overall survival of FLT3−NPM1+ AML patients in validation sets (F) Overall survival of FLT3−NPM1− AML patients in validation sets (G) Overall survival of FLT3+NPM1+ AML patients in validation sets (H) Distribution of Blast cells in PB or BM in validation sets (I) Overall survival of non-APL AML patients in validation sets according to PB blast (J) Overall survival of non-APL AML patients in validation sets according to BM blast (K) Overall survival of patients in validation sets with high PB blast (L) Overall survival of patients in validation sets with low PB blast. [file Image_1.tif]

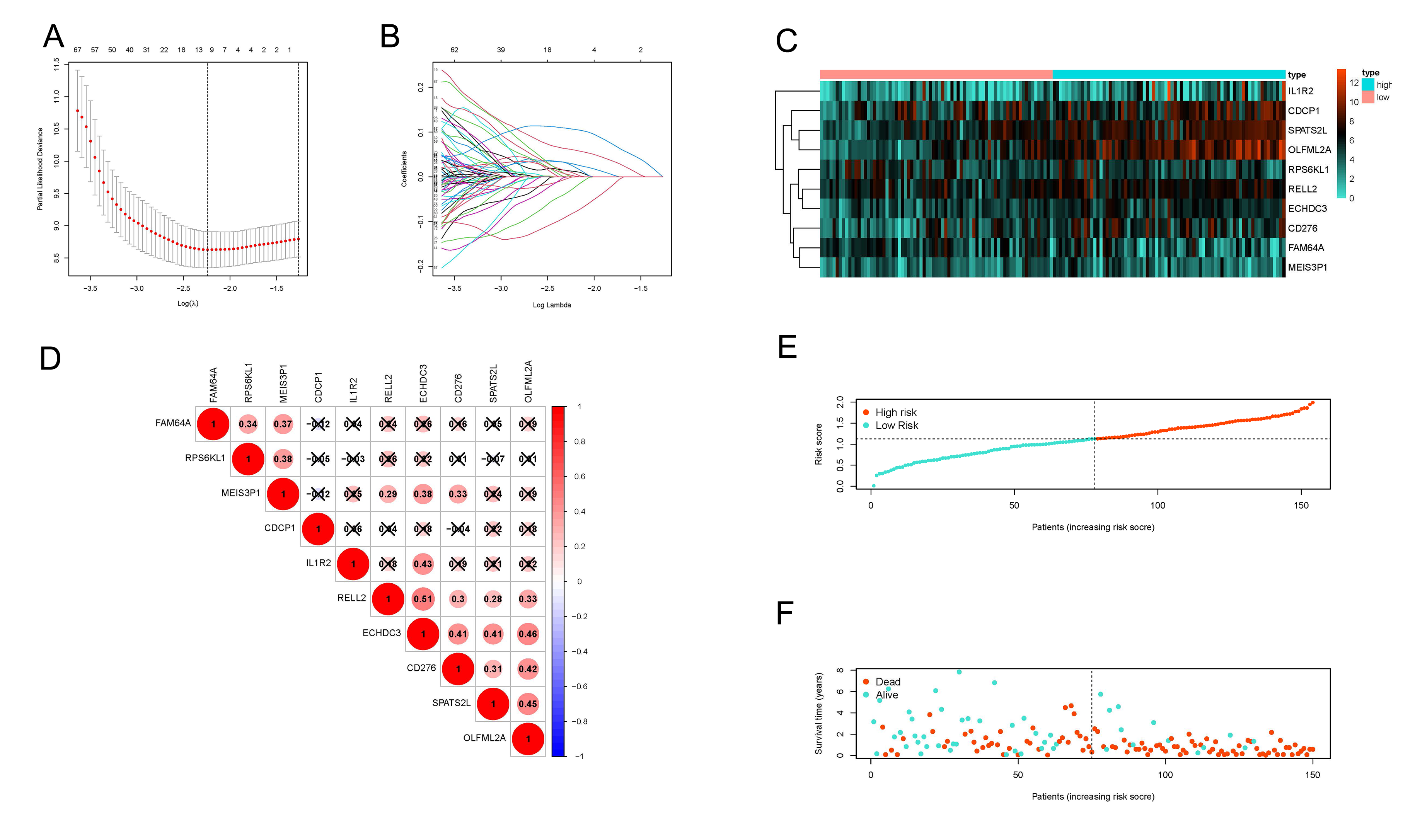

Supplement: Supplementary Figure 2 — LASSO regression (A) The interrelation of 4 key genes acquired from LASSO regression (B) The evaluation progress of gene selection in LASSO regression (C) Heatmap of 9 key genes expression acquired from LASSO regression (D) Two-way interactions checked between the factors with the main effect in Lasso regression. [file Image_2.tif]

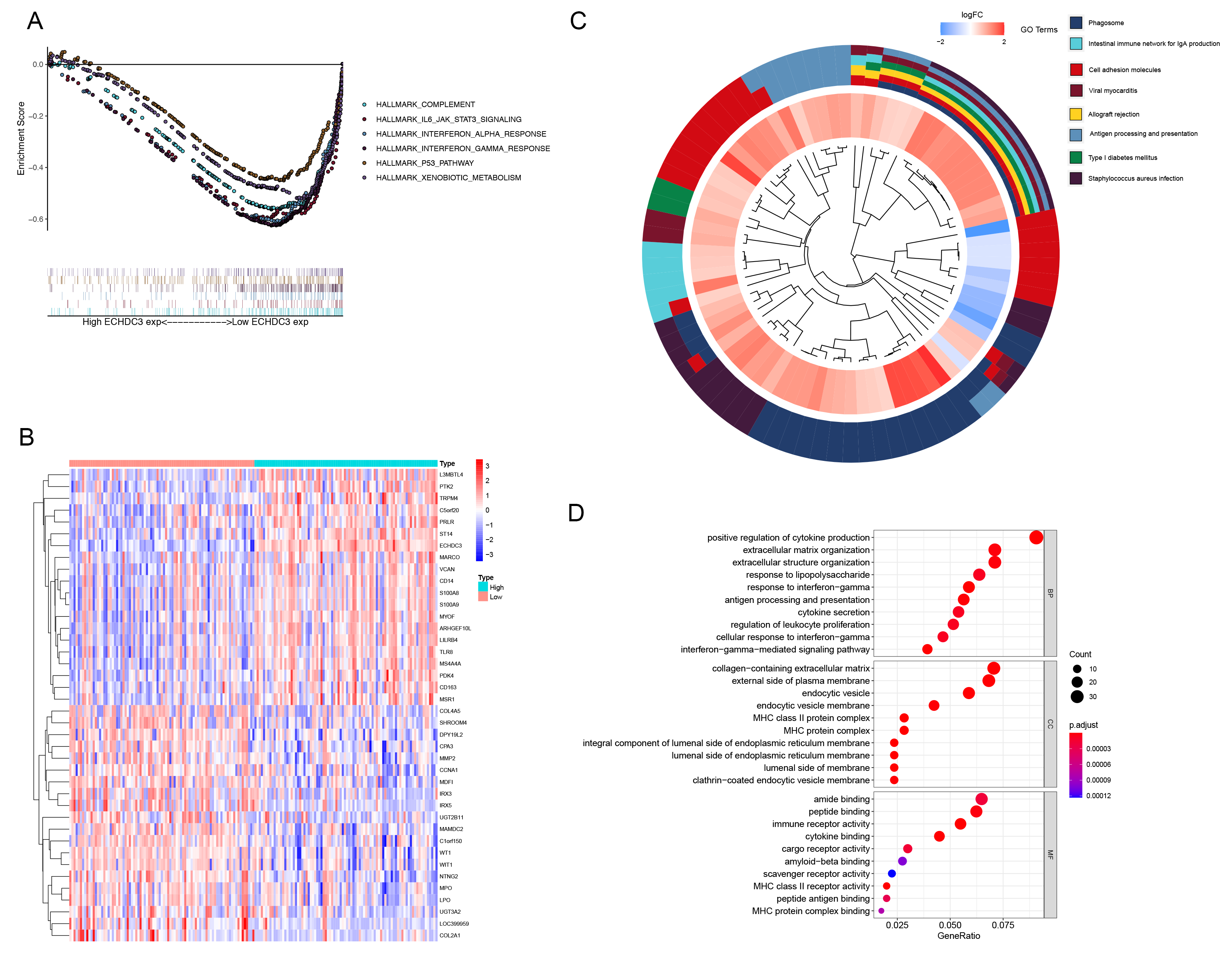

Supplement: Supplementary Figure 3 — Bioinformatic analysis of ECHDC3 function (A) Hallmark gene sets enrichment analyses for ECHDC3high and ECHDC3low AML patients by GSEA. (B) Heatmap of the deferentially expressed gene related to ECHDC3 expression. (C) KEGG Cluster plot displaying a circular dendrogram of the clustering of the ECHDC3 expression profiles. The inner ring shows the colored logFC, and the outer ring is assigned functional terms. (D) Identification of AML subtype-specific GO terms. Top 10 GO terms enriched in the Biological Process (BP), Molecular Function (MF), and Cellular Component (CC). [file Image_3.tif]

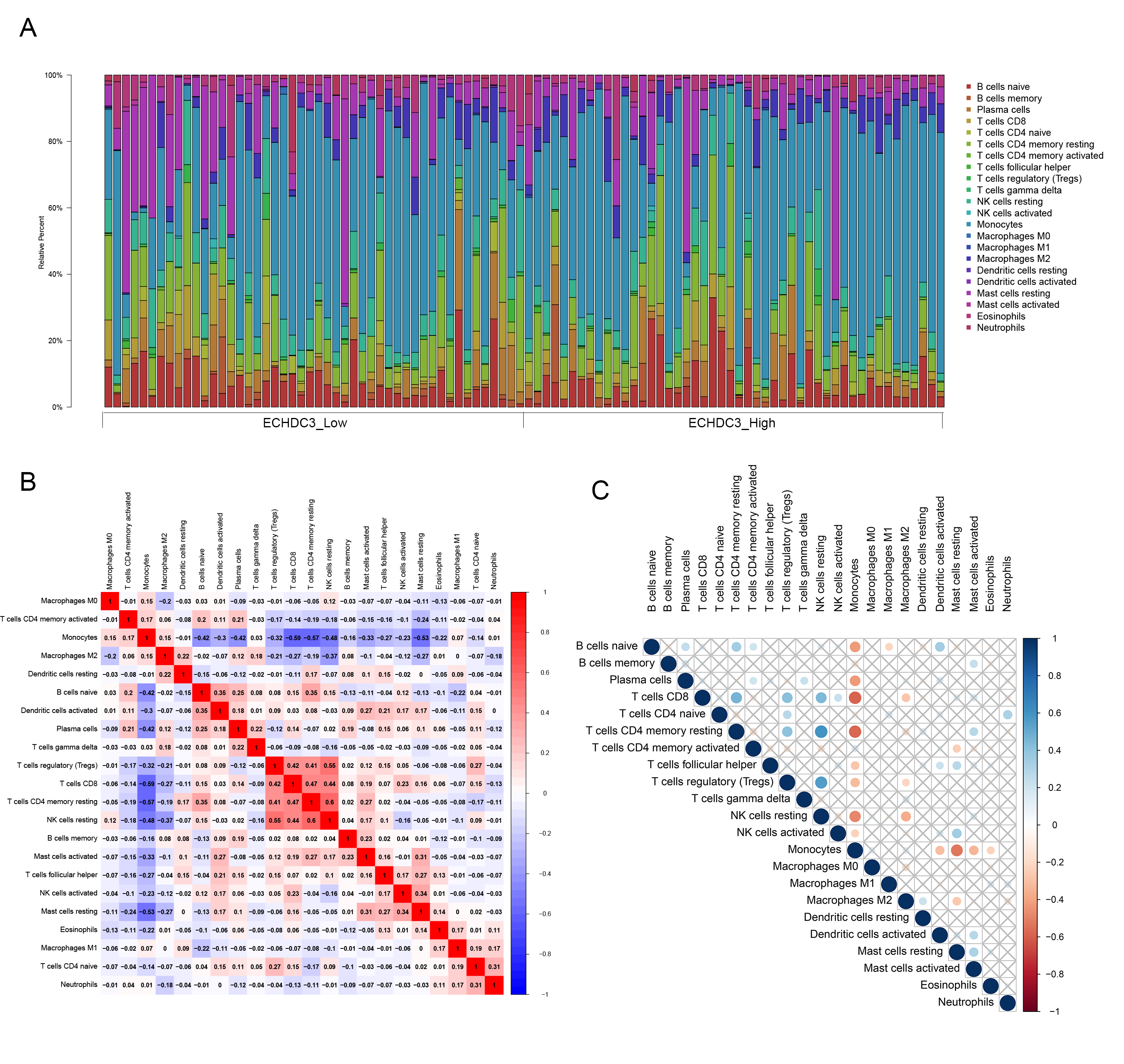

Supplement: Supplementary Figure 4 — Immune landscape between ECHDC3high and ECHDC3low groups (A) The heat map visualized the percentage abundance of tumor-infiltrating immune cells in each sample. (B) Immune cell infiltration correlation matrix. (C) Correlation matrix of all 22 immune cell proportions. [file Image_4.tif]
